# Biologics in Systemic Lupus Erythematosus: Recent Evolutions and Benefits

**DOI:** 10.3390/pharmaceutics16091176

**Published:** 2024-09-06

**Authors:** Nilima Rajpal Kundnani, Mihaela Codrina Levai, Mihaela-Diana Popa, Claudia Borza, Mihai Iacob, Alexandra Laura Mederle, Alexandru Blidisel

**Affiliations:** 1 Department of Cardiology—Internal Medicine and Ambulatory Care, Prevention and Cardiovascular Recovery, “Victor Babeș” University of Medicine and Pharmacy, 300041 Timisoara, Romania; knilima@umft.ro; 2 Research Centre of Timișoara Institute of Cardiovascular Diseases, “Victor Babeș” University of Medicine and Pharmacy, 300041 Timisoara, Romania; 3 Discipline of Medical Communications, Department 2—Microscopic Morphology, “Victor Babeș” University of Medicine and Pharmacy, 300041 Timisoara, Romania; 4 Department of Microbiology, “Victor Babes” University of Medicine and Pharmacy, 300041 Timisoara, Romania; 5 Discipline of Pathophysiology, Department of Functional Science, “Victor Babeș” University of Medicine and Pharmacy, 300041 Timisoara, Romania; claudia_borza@yahoo.com; 6 Center for Translational Research and Systems Medicine, “Victor Babeș” University of Medicine and Pharmacy, 300041 Timisoara, Romania; 7 Centre of Cognitive Research in Pathological Neuro-Psychiatry NEUROPSY-COG, “Victor Babeș” University of Medicine and Pharmacy, 300041 Timisoara, Romania; 8 Advitam Medical Center, 300150 Timisoara, Romania; 9 Faculty of Medicine, “Victor Babeș” University of Medicine and Pharmacy, 300041 Timisoara, Romania; 10 Clinic of Surgical Semiotics and Thoracic Surgery—1, Department IX—Surgery—1, Victor Babes” University of Medicine and Pharmacy, 300041 Timisoara, Romania; 11 Center for Hepato-Biliary-Pancreatic Surgery (CHBP), “Victor Babes” University of Medicine and Pharmacy, 300041 Timisoara, Romania

**Keywords:** systemic lupus erythematosus, C5 complement protein, CD20, Type-I interferon receptors, quality of life

## Abstract

Introduction: Systemic lupus erythematosus (SLE) is a multifaceted autoimmune disorder characterized by significant autoantibodies, particularly targeting nuclear antigens. SLE pathogenesis involves genetic, environmental, and hormonal factors. The disease course includes flares and remission and involves various organs. Recent therapeutic progresses, including biologics, have improved management and prognosis, though the long-term impact of novel therapies remains to be determined. Biologics in SLE: Rituximab, the earliest B-cell-oriented biologic, binds CD20 and depletes CD20+ B cells, leading to remission in some SLE patients. Belimumab is a B-cell-activating factor (BAFF) inhibitor with a recent additional indication for lupus nephritis. The CALIBRATE and BLISS-BELIEVE studies investigated combinations of these drugs with conventional therapies, showing varied efficacy. Ocrelizumab and obinutuzumab, newer CD20-oriented SLE therapies, together with ofatumumab and veltuzumab, are also promising. The latest trials highlight their efficacy and safety. Anifrolumab, targeting type-I interferon receptors, was evaluated in the TULIP 1/2 trials. The ongoing TULIP LTE trial supports the long-term safety and efficacy of anifrolumab. Additionally, the IRIS Phase III trial is exploring anifrolumab for lupus nephritis, showing favorable renal responses. Tocilizumab and secukinumab are being assessed for SLE, with mixed outcomes. Several biologics targeting the C5 complement protein, together with immunomodulators and immunotherapeutics, are also under investigation for potential benefits in SLE. Discussion: Biologics in SLE target specific immune components, aiming to improve disease control and reduce the side effects of conventional therapy. However, trial outcomes vary due to factors like inclusion criteria and trial design. Conclusions: Biotechnology progress enables targeted biologic therapies for SLE, reducing disease activity and improving patients’ quality of life.

## 1. Introduction

Systemic lupus erythematosus (SLE) is a complex autoimmune disorder that affects multiple organ systems and is characterized by periods of exacerbation and remission. It features significant titers of a variety of autoantibodies, particularly those directed against nuclear antigens. Despite extensive research, the initiating mechanisms of SLE remain elusive. The disease’s pathogenesis is multifactorial, involving a combination of genetic predisposition, environmental triggers, and hormonal influences [1]. Genetic factors may include specific gene polymorphisms that influence immune regulation, while environmental factors such as UV radiation, infections, and certain medications can precipitate disease onset or flares. Hormonal influences, particularly estrogen-related, are also thought to contribute to the higher prevalence of SLE in women [2].

SLE is a prototypical type III hypersensitivity disorder, with immune complex deposition that impacts a wide array of organs, often leading to significant morbidity. The clinical course of SLE is highly variable, with patients experiencing flares—periods of increased disease activity—interspersed with periods of relative quiescence. These flares can involve different organs and systems, including the skin, joints, kidneys, cardiovascular system, and nervous system, among others. During flares, the immune system’s attack on healthy tissues results in inflammation and damage, contributing to progressive disability and cumulative organ damage over time. The diverse and unpredictable nature of SLE flares necessitates constant monitoring and a flexible therapeutic approach to manage symptoms and prevent long-term complications [3].

SLE pathogenesis includes three key steps in which the immune system has an active and decisive contribution:

- Autoantigen presentation: dendritic cells and other antigen-presenting cells (e.g., B cells) process and present self antigens, such as nuclear antigens from apoptotic cells to autoreactive T cells. The autoantibodies against nuclear, cytoplasmic, and membrane antigens will play an important pathogenetic role. Two nuclear antigens are pathognomonic for SLE: double-stranded DNA (dsDNA) and Sm antigens from the U-1 small nuclear ribonucleoprotein complex [1].
- T cell activation: autoreactive T cells become activated and provide assistance to B cells, promoting their differentiation into plasma cells. The B-cells could be activated in a T-cell-dependent manner, as most of the target antigens for autoantibodies in SLE are proteins (Sm, Ro/SS-A, La/SS-B, RNP, histones, C1q) or in a T-cell-independent manner (for non-proteic antigens such as dsDNA) [4,5].
- Autoantibody production: activated B cells produce autoantibodies that form immune complexes with cognate antigens. The production of autoantibodies against nuclear and cytoplasmic antigens is a hallmark of SLE. Antinuclear antibodies (ANAs) are present in nearly all patients, with antibodies such as anti-dsDNA and anti-Sm being highly specific for SLE [5].

The 2023 EULAR recommendations for SLE management provide guidance on treatment choices [6]. Hydroxychloroquine (HCQ) remains the strongest choice for first-line therapy, with a typical target dose of 5 mg/kg body weight, further adjusted based on flare risk and retinal toxicity. Glucocorticoids (GCs) are recommended for maintenance, limited to ≤5 mg/day. Reducing reliance on GCs aims to use disease-modifying antirheumatic drugs (DMARDs) or DMARD biologics early to minimize GC dosage. Furthermore, GC should be withdrawn as soon as sustained remission of lupus nephritis (LN) is achieved, provided that extrarenal manifestations allow it. Alternate treatments include IV cyclophosphamide for life-threatening cases, second-line biologics for active skin disease, and mycophenolate mofetil (MMF) as an acute treatment for severe autoimmune thrombocytopenia. The second-line biologics include belimumab (BEL), anifrolumab (ANF), and rituximab (RTX), which offer additional options beyond traditional therapies. Rituximab is recommended in refractory lupus, ANF and BEL in mucocutaneous manifestations of SLE, though the latter are not recommended in patients with severe neuropsychiatric disease. BEL, a human monoclonal antibody (mAb) that inhibits BAFF, has been approved in the United States, Canada, and the European Union since 2011 for the treatment of SLE and LN in adults and children aged 5 years and older. Anifrolumab, a human monoclonal antibody that blocks the type I interferon receptor (IFN-R), was more recently approved in the United States (2021) and European Union (2022) as an add-on treatment in adults with (SLE). RTX is the biologic that is most often used off-label in SLE. Voclosporin, a novel calcineurin inhibitor, is also recommended as an add-on therapy for the management of active LN [7,8].

In the last decades, significant steps have been taken towards improving SLE prognosis, owed in no small measure to better management with both the classical therapies and the new DMARDS, small molecules, or biologics. The 5-year survival of patients with LN, traditionally between 95% and 75%, has also improved, but there is yet a need for clear evidence regarding the long-term impact of novel therapies on mortality [9].

This review aims to provide all the latest developments in the treatment regimens for SLE in one place.

SLE has been associated with abnormal development, maturation, and function of B cells. The immune dysregulation in SLE is marked by a loss of tolerance to ubiquitous self-antigens, which is sustained by complex interactions between innate and adaptive immune pathways, ultimately leading to end-organ damage. In patients with SLE, immature transitional B cells, memory B cells, and plasma cells proliferate in peripheral tissues, and autoreactive B cells are inadequately eliminated in the bone marrow by clonal deletion.

Overactive T-cell-dependent germinal center-like B cell proliferation results in an expanded and largely uncontrolled population of memory B cells and plasma cells. Once immune tolerance is overcome, autoreactive B cells can further propagate the disease through various mechanisms, including autoantibody production and immune complex deposition. Additionally, B cells contribute to the disease process by producing cytokines, presenting antigens to T cells, inducing epitope spreading, and amplifying tissue-specific autoimmune responses. These multivalent B-cell functions underscore the pivotal role of B cells in the pathogenesis and progression of SLE; therefore, the first biologicals were aimed at the B cells [5].

Both type I (IFN-α and IFN-β) and type II (IFN-γ) interferons are significantly upregulated in SLE. The “IFN signature”, with high levels of IFN-α and increased expression of type I IFN-regulated genes, is a hallmark of SLE. TNF-α is another pro-inflammatory cytokine that is upregulated in SLE. IL-1 is a pro-inflammatory cytokine that also contributes to the inflammatory environment in SLE. IL-6 is a pleiotropic cytokine that promotes the activation and differentiation of immune cells, including T cells, B cells, macrophages, and neutrophils. It is crucial in the development of systemic autoimmunity and inflammatory responses in SLE. IL-17 has been found to be elevated in SLE patients in association with disease severity. IL-10 is typically an anti-inflammatory cytokine that can paradoxically promote B cell survival and autoantibody production in SLE. Elevated levels of IL-12, a cytokine involved in the differentiation of naive T cells into Th1 cells, have been observed in SLE. IL-23, which is important for the maintenance of Th17 cells, has been implicated in the pathogenesis of SLE. BAFF is essential for B cell survival and differentiation, with elevated levels of this B cell marker being associated with increased autoantibody production in SLE [10,11,12,13].

In SLE, the membrane-bound CD95L (Fas ligand) is cleaved by metalloproteases, resulting in the soluble form, cl-CD95L. The latter promotes the migration of immune cells, particularly T cells, and acts as a chemoattractant for Th17 cells. The level of cl-CD95L correlates with disease progression [14,15].

## 2. Biologics Targeting the B Cells

The earliest B-cell-depleting biologic agent, used in the treatment of SLE for over two decades, is rituximab, a chimeric human/murine Igg1 mAb targeting CD20. CD20 is a surface antigen expressed early in B cell development, but the expression is lost on plasma blasts and plasma cells. Administration of rituximab leads to the rapid depletion of CD20^+^ B cells by antibody-dependent cellular cytotoxicity (ADCC) and complement-dependent cytotoxicity (CDC) [11,16,17]. After rituximab treatment, some patients replenish with naive B cells and the disease enters remission. In others, the B cell pool is not completely drained so they can replenish from memory B cells and could benefit from further rituximab treatment. Belimumab is a human IgG1λ monoclonal antibody that inhibits BAFF. It was FDA-approved in 2011 for the treatment of adult patients with active, autoantibody-positive SLE who are receiving standard therapy. It was also authorized for use in the European Union by the EMA in 2011 as add-on therapy in adults with SLE as an IV formulation and as a subcutaneous (SC) formulation in 2017. The SLE indication was extended to cover children for the IV formulation in 2019 [18]. The CALIBRATE trial, completed in 2017, investigated the safety and efficacy of LN treatment with a combination of RTX and cyclophosphamide (CTX) or a combination of RTX and CTX followed by treatment with BEL. The study, performed on patients diagnosed with SLE and active proliferative LN, was the first randomized, controlled trial to examine the safety and efficacy of the RTX+BEL combination in patients with LN. After 48 weeks, the results showed only one-third of the patients achieved complete response. The trial was not intended to evaluate the efficacy, but on the contrary, its aim was to mainly evaluate the safety. In this regard, the BEL add-on therapy to a rituximab+CYC treatment regimen was deemed safe in patients with refractory LN [13,19].

The BLISS-BELIEVE study, investigating adult SLE patients on a stable treatment regimen, was completed in 2021, with the first results reported in 2022. It included 292 patients enrolled in three arms: BEL+placebo, BEL+RTX, and BEL+standard therapy. For many of the study indicators, the reported results showed the best results in the treatment arm with BEL+standard therapy (including immunosuppressants, anti-malarials, NSAIDs, and/or corticosteroids tapered down to prednisone equivalent of ≤5 mg/day). The most striking differences were in the time to first severe flare (730 days for BEL+SoC vs. 379 days for BEL+RTX) and duration of clinical remission (176 days for BEL+SoC vs. 73 days for BEL+RTX). In other indicators, these two treatment arms yielded equivalent results. Also regarding BEL, the SABLE observational study has been continued since 2013, with over 3100 enrolled participants to date. The patients, adults with active, autoantibody-positive SLE, are distributed in one of two cohorts—one with participants receiving or initiating BEL+SOC at baseline and the comparison cohort with participants not receiving BEL but receiving SOC at baseline. This research aims to evaluate the incidence of adverse events of special interest (AESI) and treatment effectiveness [20,21].

In order to better characterize the pharmacokinetic profile of BEL 200 mg SC and its safety in pediatric SLE participants (aged 5 to 17 years), a multicentric open-label trial (EUCT 2023-509413-37-00, NCT04179032) was launched in 2020, using repeat doses of 200 mg BEL administered subcutaneously (SC) + SoC compared to placebo + SoC. This trial’s positive outcome resulted in the biologic obtaining approval as a 200 mg single-dose prefilled autoinjector for patients 5 years of age and older with active SLE who are receiving standard therapy.

An undergoing study, PREDICT, was launched to explore a variant of the *TNFSF13B* gene, commonly known as *BAFF-var*, that is linked to an elevated risk of developing immune-mediated diseases, including SLE and rheumatoid arthritis (RA). The polymorphisms of this gene result in increased production of BAFF, which is associated with more severe disease manifestations. In SLE, higher BAFF levels correlate with elevated anti-Sm and anti-dsDNA antibody titers, complement consumption, and a heightened risk of disease flares. Similarly, in RA, this variant is associated with higher disease activity. This could also be one explanation for the heterogeneous responses to BEL in previous research [22,23].

Previous developments in BAFF-targeting include tabalumab (TAB), a fully human, subcutaneously administered IgG4κ monoclonal antibody that binds and neutralizes both membrane-bound and soluble BAFF. Its safety and efficacy were evaluated in ILLUMINATE-2, a 52-week, phase III, multicentre, randomized, double-blind, placebo-controlled study. The study enrolled 872 patients that completed the 52-week trial, randomized in 3 arms: (1) Q2W receiving TAB every 2 weeks and SoC, (2) Q4W receiving TAB alternating with placebo every 2 weeks plus SoC, or (3) placebo Q2W plus SoC. The primary endpoint was met, as the proportion of patients achieving SLE Responder Index 5 (SRI-5) improvement at week 52 was significantly higher with the 120 mg every 2 weeks (120 Q2W) dosing regimen (38.4%) compared to placebo (27.7%). This was the first time a BAFF inhibitor achieved that efficacy measure in a primary endpoint. In regards to adverse events, depression and suicidal ideation, although rare, were more commonly reported with tabalumab compared to placebo. Later, due to negative results in another phase III trial, tabalumab development was halted [24].

Epratuzumab is an IgG1κ humanized monoclonal antibody targeting CD22, a cell surface glycoprotein found on mature B-cells and various malignant B-cells. It received FDA orphan designation for the treatment of non-Hodgkin’s lymphoma (the designation was withdrawn in 2019). The EMBLEM open-label extension study evaluated the long-term effects of epratuzumab treatment in adult patients with moderate-to-severe SLE. Compared to the 12-week, double-blind EMBLEM study, the open-label extension identified no new safety or tolerability signals, and epratuzumab was well-tolerated over two years, starting in 2013. The disease activity reduction was maintained over two years relative to EMBLEM baseline, and there was a steroid-sparing effect—patients receiving epratuzumab showed decreased GC use (for those receiving >7.5 mg/day). The ALLEVIATE 1 and 2 trials evaluating epratuzumab for moderately-to-severely active SLE were discontinued prematurely due to an interruption in drug supply. Exploratory analyses showed that epratuzumab had a response rate of 44.1% (360 mg/m²) and 20.0% (720 mg/m²) at week 12, compared to 30.0% for placebo [25,26]. Despite the discontinuation, the initial efficacy and safety profile supported further development of epratuzumab for SLE treatment. No further trials of this promising biologic were identified.

Ocrelizumab is an IgG1κ humanized monoclonal anti-CD20 antibody, part of the ongoing REGENCY Phase III trial in renal SLE, started in 2020. It is also being investigated in non-renal SLE as part of the ALLEGORY Phase III trial, which started in 2021. Unlike obinutuzumab, ocrelizumab’s trial in SLE was halted due to an increased rate of opportunistic infections in the arm with ocrelizumab in combination with standard of care (versus standard of care alone). However, ocrelizumab is approved for multiple sclerosis (MS) treatment, showing efficacy in relapsing-remitting MS and primary progressive MS. It is thought to trigger ADCC and CDC against B cells. Obinutuzumab, licensed for hematological malignancies, is a next-generation humanized type-2 anti-CD20 antibody with enhanced ADCC by glycoengineering and is capable of inducing direct cell death. In a study evaluating SLE patients with secondary non-response to RTX (2NDNR), obinutuzumab was found to be effective and steroid-sparing, with the following key effects: significant reductions in disease activity measured by SLEDAI-2K (SLE Disease Activity Index) and BILAG-2004 (British Isles Lupus Assessment Group) scores, improvement in complement C3 and reduction in anti-dsDNA titers, some patients achieved LLDAS (Lupus Low Disease Activity State), also peripheral B cell depletion was observed. Thus, among the agents targeting CD20, obinutuzumab appeared promising for SLE patients with secondary non-response to rituximab [27,28,29,30]. A later phase III trial (EUCT 2023-504774-38, NCT04963296) aims to investigate the efficacy and safety of obinutuzumab in SLE by assessing the impact of obinutuzumab on disease activity and including measures such as clinical response and autoantibody levels as secondary endpoints.

### 2.1. Combined Strategies against B Cells, Bispecific Antibodies, and Trogocytosis

Other biologics targeting CD20 with the potential to be useful in SLE are ofatumumab and veltuzumab. Ofatumumab, a fully human IgG1κ monoclonal antibody, was FDA-approved for use in adults with MS in 2020 and EMA-approved in 2021. This biologic was tested in ASCLEPIOS I/II trials and ALITHIOS extension, proving fewer relapses compared to placebo, reduced risk of disability worsening at three and six months, and less lesion activity on MRI scans. In the ALITHIOS open-label extension (up to 6 years, still ongoing), recently diagnosed, treatment-naïve patients with relapsing MS experienced 44% fewer relapses, showed significant reductions in MRI lesions (Gd+ T1 and neT2), and had fewer 3- and 6-month confirmed disability worsening events. The drug also maintained a similar safety profile over the treatment period (up to 6 years), with no new safety risks identified. In SLE, ofatumumab was only employed in small-scale off-label applications, with success (B-cell depletion) in 12 out of 16 patients previously allergic to RTX and in two cases of early-onset juvenile SLE with thrombocytopenia, also presenting severe reactions to RTX [31,32,33,34,35]. Currently, there are no active clinical trials investigating the safety and efficacy of ofatumumab in SLE. Veltuzumab is a 90% humanized IgG1κ monoclonal antibody targeting CD20, having been granted orphan drug status by the FDA in 2015 for immune thrombocytopenia. This biologic was investigated in the VELVET trial for rheumatoid arthritis, but the trial was terminated by voluntary halt, without any conclusions being inferred from the data on 11 participants treated. Individually, both epratuzumab and veltuzumab trigger ADCC and CDC against B cells. In SLE therapy, a bispecific hexavalent antibody combining epratuzumab and veltuzumab shows enhanced trogocytosis. Trogocytosis is an interaction typically occurring between immune cells, such as T cells, B cells, natural killer cells, and antigen-presenting cells (APC), whereby one cell takes over and expresses or internalizes portions of the membrane and surface molecules from the APC. This effect leads to significant reductions in B-cell surface markers such as CD19, CD20, CD21, CD22, CD79b, CD44, CD62L, and β7-integrin, when compared to epratuzumab and veltuzumab administered individually. This approach results in substantially less immunocompromising B-cell depletion than what is typically observed with anti-CD20 mAbs like veltuzumab or RTX, whether used alone or in combination with epratuzumab. Additionally, a CD22/CD19 bispecific hexavalent antibody, which enhances trogocytosis of certain antigens with minimal B-cell depletion, may also have therapeutic potential. This bispecific antibody is a promising candidate for improved treatment of SLE, offering significant advantages over the combination therapy of the two parental antibodies [36].

Mosunetuzumab is a humanized IgG1 monoclonal bispecific antibody targeting CD20 and CD3 and thus engaging T cells to eliminate B cells by facilitating ADCC. It was approved in 2022 by the FDA and EMA for the treatment of adults with relapsed or refractory follicular lymphoma after two or more lines of systemic therapy [37]. A trial (EU ID 2023-509188-26-00) is currently underway to evaluate the safety, tolerability, pharmacokinetics, and pharmacodynamics of subcutaneously administered mosunetuzumab to participants with SLE.

### 2.2. Biologics Targeting IFN-Mediated Mechanisms

ANF is an IgG1κ monoclonal antibody that binds to the type I IFN-R, blocking the activity of type I IFNs (such as IFN-α and IFN-β). The most recent clinical trials for ANF were part of the TULIP (Treatment of Uncontrolled Lupus via the Interferon Pathway) program. These Phase III trials, known as TULIP 1 and TULIP 2, evaluated the efficacy and safety of ANF compared to placebo in patients with moderately-to-severely active autoantibody-positive SLE who were receiving standard care treatment. Although TULIP-1 did not meet its primary endpoint, the results from MUSE and TULIP-2 trials indicate that administering ANF for up to 48 weeks can lead to clinical and immunological improvements in SLE patients with predominantly high type-1 *IFN* gene signatures. Anifrolumab is currently undergoing a long-term extension (LTE) of the pivotal TULIP Phase III program (2016–2020). The TULIP LTE trial evaluated the safety and efficacy of ANF over a 3-year period in patients with SLE. During this extension, patients received either ANF 300 mg or placebo every 4 weeks, in addition to standard care treatment. The results showed that ANF is safe and effective when used long-term for treating SLE, with an exposure-adjusted incidence rate (EAIR) per 100 patient-years less than placebo and non-opportunistic serious infections comparable between groups (EAIR of 3.7 with ANF versus 3.6 with placebo) [38].

The TULIP 1 trial was a 52-week, placebo-controlled, multicenter study. Patients received intravenous ANF (300 mg in TULIP-1 and TULIP-2; 150 mg in TULIP-1 only) or placebo every 4 weeks alongside standard therapy. The design included mandatory attempts to taper GCs. The primary endpoint was the SLE Responder Index (SRI-4) at Week 52, but the trial did not achieve statistical significance for this endpoint. Despite missing the primary endpoint, ANF 300 mg showed numeric improvements relative to placebo in skin disease activity, joint disease activity, and overall disease activity. The TULIP 2 trial demonstrated a meaningful reduction in disease activity compared to the placebo. Specifically, at the end of the four-year TULIP program (Week 208), 30.3% of patients treated with ANF achieved remission, compared to 18.3% in the SoC group [39].

The IRIS Phase III trial (NCT05138133, EUCT 2023-506359-68-00) is a significant clinical study evaluating ANF in active proliferative LN. Up to 360 patients aged 18 to 70 are expected to participate, receiving intravenous ANF as an intensified regimen (IR): Initially 900 mg followed by 300 mg every 4 weeks, basic regimen (BR): 300 mg every 4 weeks or placebo, administered alongside SoC (MMF and GC). The results so far demonstrate that more patients in the IR achieved complete renal response at Week 104 compared to BR or placebo; the IR group also achieved sustained GC tapering and there were numerical improvements of estimated glomerular filtration rate observed in both ANF groups. The safety profile through Year 2 was consistent with Year 1. The encouraging efficacy results support further investigation of ANF in larger populations with active proliferative LN.

An ongoing, currently recruiting multicenter, randomized, double-blind, placebo-controlled, phase 3 study (NCT02446899—completed, EUCT 2024-513031-24-00—currently recruiting) evaluates the efficacy and safety of subcutaneous ANF in adult patients with SLE. This study aims to assess the effects of ANF administered subcutaneously (s.c.) compared to placebo. Participants have moderately to severely active, autoantibody-positive SLE while receiving standard of care.

An immunotherapeutic vaccine phase 2b trial on 185 recruited SLE patients was aimed at inducing anti-IFN-α2b serum antibodies and was recently completed. Successful induction of neutralizing antibodies was achieved in 91% of treated patients, with reduced *IFN* gene signature. The disease activity score in treated patients was also lower, with a significant CS sparing effect. This interferon-α kinoid (IFN-K) vaccine is a promising candidate for lowering pro-inflammatory IFN-mediated responses in SLE [40,41].

### 2.3. Biologics Targeting IL-6 Mediated Mechanisms

Tocilizumab (TCZ) is a humanized IgG1κ monoclonal antibody that targets the interleukin-6 receptor (IL-6R). TCZ binds to both the soluble and membrane-bound IL-6 receptors, preventing the cytokine from interacting with its receptor and thereby inhibiting its inflammatory effects. In the European Union, this biologic is approved for the treatment of rheumatoid arthritis, systemic juvenile idiopathic arthritis, juvenile idiopathic polyarthritis, giant cell arteritis, cytokine release syndrome, and COVID-19, while in the USA, it is approved for all of the above plus systemic sclerosis-associated interstitial lung disease. It has been used off-label in SLE, with success, according to a number of recent case reports. In one case, TCZ induced a prompt recovery of SLE-associated pleural effusion in an adolescent; afterwards, the patient remained on maintenance TCZ without relapses for up to 1 year. In a later case of a 20-year-old female, a partial remission was obtained for treatment-resistant lupus, with resolution of fever and arthritis and partial resolution of nephritis. Two other female SLE patients with fever and elevated CRP also responded to TCZ treatment, with resolution of fever and normalization of inflammatory markers. All these elements underline the importance of targeting IL-6-mediated inflammatory pathways in SLE, especially in patients with elevated IL-6 and CRP levels and uncontrolled disease despite first-line therapies. There is a risk of neutropenia associated with TCZ use, as IL-6 mobilizes neutrophils, and TCZ could bind to the neutrophils IL6R, exposing them to phagocytosis [42,43,44,45].

### 2.4. Biologics Targeting IL-17 Mediated Mechanisms

Secukinumab (SEC) is a human IgG1κ monoclonal antibody used to treat several inflammatory conditions, such as plaque psoriasis, psoriatic arthritis, ankylosing spondylitis, and enthesitis-related arthritis. It binds to IL-17A, blocking its interaction with IL-17 receptors on the surface of target cells and thus prevents the pro-inflammatory effects mediated by this cytokine. This biologic was authorized for use in the European Union (EU) in 2015 and FDA-approved for adults with psoriatic arthritis, ankylosing spondylitis, and non-radiographic axial spondyloarthritis in 2023. A recent report outlines a case of drug-induced lupus with renal involvement where SEC was reintroduced after 3 months pause due to the COVID-19 pandemic in a patient with psoriatic arthritis, previously undergoing treatment with the biologic for 2 years. After achieving lupus remission with conventional therapies—prednisolone+MMF+HCQ—the patient was switched to guselkumab (an IL-23 inhibitor) with favorable outcome on psoriasis skin response [46]. In a similar fashion, a case is described where a patient developed secukinumab-induced SLE while treated for ankylosing spondylitis. SEC was withdrawn, and the biologic and clinical signs of lupus were remitted within 15 months. Adalimumab (TNF alpha inhibitor) was initiated in the patient, with good clinical response of the arthritis and no relapse of SLE [47]. However, another case was described involving a patient with pre-existing psoriasis associated with SLE, which is a clinically rare entity. In this case, after 5 weeks of treatment with SEC 300 mg/wk, there was a marked improvement in both biological markers of inflammation and disease activity scores (PASI and SLEDAI, respectively) [48]. Secukinumab can increase the risk of infections, especially in patients with pre-existing chronic infections or a history of relapsing infections. The biologic was also considered a potential trigger of inflammatory bowel disease, but a recent real-world study on 306 secukinumab-treated patients did not provide evidence in this direction. However, an increased rate of gastrointestinal-related adverse events was noted [49].

Another anti-IL-17 agent is ixekizumab, a humanized IgG4κ monoclonal antibody with similar indications to SEC. However, the therapeutic targeting of IL-17A with ixekizumab has not been documented in clinical practice for lupus. Bimekizumab is a humanized IgG1κ monoclonal antibody that binds IL-17A and IL-17F. It was initially authorized for the treatment of moderate to severe plaque psoriasis in adults and also approved for moderate-to-severe hidradenitis suppurativa in 2024 by the FDA. Its use is also not documented in SLE [50].

### 2.5. Biologics Targeting IL-12/IL-23-Mediated Mechanisms

Ustekinumab (USK) is an anti-IL-12/IL-23, IgG1κ human monoclonal antibody, approved in 2009 by the FDA and EMA for the following indications: plaque psoriasis, psoriatic arthritis, and Crohn’s disease. Ustekinumab was demonstrated to be a safe and effective treatment in adult patients with active SLE over a two-year period in an extension of a phase II clinical trial. The researchers evaluated multiple indicators of disease improvement and found that the medication was associated with clinical improvements in both overall and organ-specific disease activity. Of the original 102 participants enrolled in the initial phase II trial, 46 completed the long-term extension study. Some patients did not join the study extension as they had already completed their study participation. Among those who did, 24 individuals received USK from the beginning of the trial (the “USK group”), while 14 initially received a placebo (without expected therapeutic effect) and then switched to USK treatment at week 24 (the “crossover group”). There was a benefit from sustained long-term treatment in both groups: at least a 4-point improvement from their baseline disease activity scores. Additionally, 79% of the USK group and 93% of the crossover group achieved at least a 30% improvement in their physician global assessment scores from baseline, a measure of overall disease activity based on a clinician’s evaluation. Furthermore, 86% of the USK group and 91% of the crossover group experienced at least a 50% improvement in joint pain and inflammation, while 79% of the USK group and 100% of the crossover group showed at least a 50% improvement in their skin disease activity scores. Upper respiratory tract infection, urinary tract infection, and nasopharyngitis were the most common adverse event among the 102 patients [51].

### 2.6. Biologics Targeting the Complement System

Eculizumab is a fully humanized IgG2/4k monoclonal antibody that functions as a terminal complement inhibitor by binding to complement protein C5, preventing intravascular hemolysis [52]. It is the first drug approved for the treatment of complement-mediated diseases. Common adverse reactions include headache, nasopharyngitis, back pain, and nausea. It was first authorized in the USA for the treatment of patients with paroxysmal nocturnal hemoglobinuria (PNH) in 2007 and in the EU in 2017 for adults with generalized myasthenia gravis. Its use in SLE is not standard, but it was employed in patients with both SLE-associated thrombotic microangiopathy and hematopoietic stem cell transplantation-associated thrombotic microangiopathy [53]. When eculizumab was used in SLE-associated thrombotic microangiopathy, favorable outcomes were reported in 93% of the reviewed patients, while 10% reported adverse effects attributable to the biologic (nausea) or to an unknown cause (pancreatitis) [54]. Eculizumab treatment raises the possibility of type B insulin resistance development and/or sepsis, with fatal outcome. This warrants caution when the biologic is used in patients with a secondary immune deficiency and/or glucose metabolism imbalance, with close monitoring for signs of infection and prompt, efficient antimicrobial treatment [55].

### 2.7. Novel Agents Developed against Complement System Components

Ravulizumab is an IgG2/4k humanized monoclonal antibody that also targets the complement C5 protein. It was approved by the FDA in 201, for treating paroxysmal nocturnal hemoglobinuria (PNH) and atypical hemolytic uremic syndrome (aHUS) in both children and adults and by the EMA in 2019 for the same indications [56,57]. This biologic potential in SLE is being investigated in adult participants with proliferative LN or immunoglobulin A nephropathy (IgAN).

An active phase 2 study employs KP104, fusion protein, which is designed to modulate the complement system. It combines a humanized anti-C5 mAb with the functional domain of complement regulator factor H. With this dual-approach mechanism of action, it is the only drug designed to simultaneously block the upstream Complement Alternative Pathway (by the Factor H moiety) and downstream Complement Terminal Pathway (by the variable Ig domain binding C5). The open-label study, initially estimated to start in 2023, targets thrombotic microangiopathy secondary to SLE in patients with decreased platelet count, abnormal renal function, and evidence of microangiopathic hemolytic anemia [58].

### 2.8. Other Biologic Therapies Developed for SLE

Litifilimab (BIIB059), a monoclonal antibody targeting BDCA2—a plasmacytoid dendritic cell-specific antigen—is currently being investigated for the treatment of SLE and cutaneous lupus erythematosus (CLE). The LILAC study, a phase II randomized controlled trial, started in 2016. This study evaluated litifilimab in patients with SLE with active cutaneous disease or cutaneous lupus with/without SLE, all without active LN. The trial demonstrated the biologic’s superiority (in 2 treatment arms with different doses) over placebo based on skin-directed outcome measures (CLASI score) in up to 20 weeks of treatment (7 doses). Litifilimab also significantly reduced the number of swollen and tender joints in SLE patients over six months [59,60,61]. A phase III study, TOPAZ-1 (NCT04895241, EUCT 2023-505696-74-00), currently active, evaluates the efficacy and safety of litifilimab in SLE patients with active disease by enrolling an estimated 540 patients worldwide, with a year of treatment with litifilimab or placebo alongside standard lupus care.

An undergoing multicenter, randomized, double-blind placebo-controlled phase 2 study started in 2021 with 228 recruited SLE patients without severe, progressive, or uncontrolled renal disease. Nipocalimab is a fully human, nonglycosylated IgG1 mAb engineered to selectively bind to and block the IgG binding site on the endogenous neonatal Fc receptor (FcRn). By targeting FcRn, nipocalimab has the potential to treat SLE through reducing levels of pathogenic IgGs and immune complexes [62].

Another phase 2 study started in 2021 and investigated the efficacy and safety of efavaleukin alfa (AMG592) in adult subjects with active SLE, without LN or CNS lupus. Efavaleukin alfa is an interleukin-2 mutein Fc fusion protein that selectively promotes the expansion of regulatory T cells (Treg). A single dose given in healthy subjects of efavaleukin alfa has demonstrated a high Treg selectivity with a minimal change in other immune cell types. The trial in SLE patients was stopped prematurely because it met pre-defined criteria for futility [63,64].

A currently promising Immunotherapeutic approach in SLE involves KYV-101, an investigational therapy designed for patients with B cell-driven autoimmune diseases. This product is based on a human anti-CD19 chimeric antigen receptor (CAR) T-cell therapy. It aims to treat autoimmune conditions by targeting B cells, which play a role in autoimmune responses. By depleting B cells, it would reset the immune system and reduce autoimmune activity. Several clinical trials of this biologic therapy have been recorded: KYSA-1 Trial (U.S. identification NCT05938725)—an ongoing phase 1 trial investigating KYV-101 in adults with active LN, KYSA-3 trial (Germany, EU identification 2024-513329-22-00)—an ongoing phase 2 trial evaluating KYV-101 in the same patient population and KYSA-5 trial—a recently cleared phase 2 study for diffuse cutaneous systemic sclerosis (scleroderma) patients. Results from these trials are not yet available. Several other trials, with incomplete data available, are undergoing or recently finalized, with data summarized in Table 1. 

## 3. Discussion

There are undeniable clinical benefits brought forth by the use of biologics in SLE. Firstly, as the biologics are targeting and modulating specific components of the immune system implicated in SLE pathogenesis, this targeted approach can more precisely control disease activity with potentially fewer side effects compared to broad-spectrum immunosuppressants. Yet this is also a drawback, as not all mechanistic-based approaches are feasible in real-life applications, as proven by the failed biologic trials in SLE, even with an apparently sound reasoning. Amid the issues influencing the outcome of a clinical trial, one can count the inclusion criteria (which have been mainly defined by clinical disease features), outcome measures (which are usually composite clinical scores), sample size, and power, which are sometimes insufficient, careful definition of endpoints, SOC therapy, which can sometimes mask or influence the target drug effects and trial length [66].

Among the direct clinical benefits, multiple biologic agents have demonstrated significant therapeutic impact on SLE outcomes, translated as improvements in the SLE Disease Activity Index (SLEDAI), Systemic Lupus Erythematosus Responder Index (SRI), British Isles Lupus Assessment Group (BILAG) index and BILAG-Based Composite Lupus Assessment (BICLA), as well as combined/partial renal remission (CRR/PRR). Furthermore, some biologics, such as ANF and BEL, have the potential to decrease GC dependence. The reduced steroid burden will lead to a significant reduction in long-term side effects such as hypertension, osteoporosis, and increased infection risk [67].

Biologics have also been shown to reduce the frequency and severity of disease flares, thus fewer and less severe flares would lead to a more stable disease course, reducing the cumulative burden of SLE and improving long-term prognosis [68].

Patients on biologic therapy often report better quality of life outcomes, including improvements in fatigue, pain, and physical functioning. This is an important effect for promoting adherence, as well as a critical goal in managing chronic conditions like SLE, as it encompasses not only objective (physical) health but also subjective (psychological) and social well-being [69].

## 4. Conclusions

Progress in biotechnology has led to a large variety of B-cell-directed therapies, together with other immune cell-oriented therapies in SLE. In contrast to general immunosuppressants, novel treatments that interfere with specific facets of B-cell functions create the possibility of developing targeted therapeutic approaches for specific subpopulations of lupus patients. By targeting specific immune pathways, biologics offer a more focused and potentially safer treatment option, leading to reductions in disease activity, steroid use, and flare frequency while improving the quality of life for patients. These benefits underscore the significance of continued research and development in biologic therapies for autoimmune diseases and SLE in particular.

## Figures and Tables

**Table 1:** Various biologics under development for SLE.

Drug Tested	Drug Target	Target Population	Phase	Start	Current Status	Trial ID (USA/EU)
DS-7011a monoclonal antibody, daxdilimab	TLR7 antagonist	Adults with SLE, active cutaneous lupus, without active LN.	1b/2	2023	Recruiting	NCT05638802
VIB7734 monoclonal antibody	Ig-like transcript 7 on pDCs	Adults with active SLE, without active LN.	2	2021	Completed	NCT04925934
OMS721human monoclonal antibody, narsoplimab	Mannan-binding lectin-associated serine protease-2 (MASP-2)	Adults with IgA nephropathy or LN, MN, and C3 glomerulopathy, including dense deposit disease on kidney biopsy. Recently studied for transplantation-associated thrombotic microangiopathy [65].	2	2016	Unknown	NCT02682407
VAY736ianalumab	BAFF-R	Participants with active SLE.	3	2023	Authorized, not started	EUCT 2023-508499-12-00 NCT05624749
	BAFF-R	Adolescent and adult participants with moderate-to-severe disease activity and antinuclear antibody (ANA)-positive SLE.	3	2023	Recruiting	EUCT 2023-505929-14-00 NCT06133972
Human monoclonal antibodydaxdilimab	IL-17	Adults with moderate-to-severe primary discoid lupus erythematosus.	2	2023	Recruiting	EUCT 2023-509746-35-00 NCT055912222
	IL-17	Adults with active SLE, open-label extension of the RECAST SLE study.	2	2023	Active	NCT054308541
YTB323 autologous CD19-directed CAR-T cell therapy	CD19-positive B cells	Participants with severe, refractory systemic lupus erythematosus (srSLE).	1 and 2	2023	Active	EUCT 2023-510081-27-00 NCT05798117
CD19-Targeted NEX-T CAR T Cells	CD19-positive B cells	Participants with Severe, Refractory SLE	1	2023	Active	EUCT 2023-503823-24 NCT05869955
